# Analysis of Death and Survival Factors Associated with Childhood Bacterial Meningitis at a Reference Pediatric Hospital in Antananarivo, Madagascar

**Published:** 2018-07-02

**Authors:** Sedera Aurélien Mioramalala, Rado Malalatiana Ramasy Razafindratovo, Ando Rakotozanany, Raharizo Miarimbola, Goitom Weldegebriel, Jason M Mwenda, Annick Lalaina Robinson

**Affiliations:** 1 Public Health Department, Faculty of Médicine, Antananarivo, Madagascar; 2 National Malaria Country Program, Public Health Ministry, Antananarivo, Madagascar; 3 Mother and Child Department, Faculty of Medicine, Antananarivo, Madagascar; 4 Center Hospital Academic Mother Child, Public Health Ministry, Centre Hospitalier Universitaire Mère Enfant Tsaralalàna (CHU MET), Antananarivo, Madagascar; 5 WHO Inter-Country Support Team: East and Southern Africa (WHO IST/ESA); 6 WHO Regional Office for Africa (WHO/AFRO), Brazzaville, Congo

**Keywords:** Bacterial Meningitis, Childhood, Death, Madagascar, Survival Analysis

## Abstract

**Background:**

Bacterial meningitis (BM) remains a global public health problem and most cases and deaths occur in Sub-Saharan Africa and especially in children less than five years old, due to a variety of factors. This study was conducted to determine the principal factors associated with death and survival of children due to BM in a typical African tertiary health facility.

**Methods:**

A retrospective case-control study of children hospitalized for BM was conducted in the University Hospital of Tsaralalàna (CHUMET). All children aged 3 to 59 months hospitalized for bacterial meningitis and confirmed by bacteriology were included. The cases were children who died from BM, and the controls were the survivors. Data was analyzed using Stata 13.

**Results:**

The factors associated with death were the number of siblings over 3 (14,48 [2,53 - 82,95]), overcrowding (9,31 [1,39 - 62,29]), time before hospitalization of more than five days (9,26 [1,36 – 62,92]), impaired consciousness (47,74 [6,24 - 364,96]), and meningococcal meningitis (36,68 [1,90 – 704,97]).

**Conclusion:**

These factors are mainly indicators of low socioeconomic status, clinical severity of signs and particularly virulent organisms. The early detection of patients at risk allows clinicians to give them appropriate care right from admission. Further studies are necessary especially, the evaluation of the emergency care provided.

## Introduction

Bacterial meningitis (BM) is a severe infection of the central nervous system, with short-term prognosis^1^. It remains a global public health problem even though the morbidity and mortality related to this disease has significantly reduced through the use of effective vaccines against the specific causative agents. Sub-Saharan Africa bears more than 80% of the global burden of BM with nearly 500 000 deaths recorded in 2014, of which 23.37% were children under 5 years^1^–^4^. The most common causative organisms of BM worldwide, *Streptococcus pneumoniae, Nesseiria meningitidis*, and *Haemophilus influenzae* b type^3^–^6^. Knowledge of the survival predictive factors allows a more appropriate management of the disease^7^. There is a paucity of data on the survival of children with BM in Madagascar, even at the central reference hospital. The aim of this study was therefore to analyze the factors associated with death and survival from BM in children.

## Materials and Methods

### Study site

The study was conducted at the Mother and Child University Hospital of Tsaralalana (CHUMET), a reference hospital located in the city center of Antananarivo, Madagascar.

### Study type

The design was a retrospective case-control of children hospitalized for BM in CHUMET from January 2012 until December 2015.

### Population and sampling

Children aged 3 to 59 months hospitalized for BM and confirmed through a positive bacteriological examination (direct identification of bacterial pathogens through examination of cerebrospinal fluid and / or positive culture and / or positive soluble antigen) were included.

Cases are the children who died from BM and the children who survived served as controls. The controls were randomly selected, with two controls for one case.

### Data collection

Data collection was done using a pre-established case investigation form, supplemented from clinical information records when reviewing patient records, and information from the CHUMET’s laboratory.

The variables studied were: demographic and social information (age in months, gender, number of siblings), overcrowding (big family, institutional day care stay), vaccination status (according to the Malagasy Expanded Program on Immunization), the duration of illness before admission (time between the onset of signs and hospitalization), clinical presentation (temperature, condition, coma score of 1 to 5 on the Glasgow scale side, seizures, meningeal syndrome), nutritional status (assessed by waist on weight ratio), the results of bacteriological examination (direct CSF examination, culture, soluble antigen) and clinical chemistry analysis of the cerebrospinal fluid (CSF glucose and CSF protein), the duration of hospital stay and treatment outcome (living, deceased).

### Data analysis

Data was analyzed using Stata 13 statistical package. The Chi squared test, Pearson or Fisher’s exact estimate, were used for comparison of proportions, with a significant difference accepted if p <0.05. The association between BM mortality and the variables were estimated by odds ratios with 95% confidence interval. The multivariate analysis of factors associated with death was made using a multiple logistic regression model. Survival was estimated by Kaplan-Meier curves. Comparison of survival curves according to the risk factors was made by a log-rank test.

### Ethical issues and confidentiality

This study was conducted after the approval of the head of the CHUMET. The captured data were anonymized and names delinked from the data. Confidentiality and privacy of all patients and their families as well as of medical and professional staff were respected.

## Results

During the four year study period, 13073 children less than 59 months were hospitalized at the CHUMET. The number of children with BM among those hospitalized were 446, giving a prevalence of 3, 41%. Of these 446 cases of BM, there were 79 deaths, resulting in a mortality of 0, 6% and a case fatality rate of 17, 71%. The children with incomplete data and/or less than 3 months of age and/or no immunization card were excluded. Our study population became 96 children from 3 to 59 months old, of which 32 represented cases and 64 the controls.

### Univariate analysis

In our study, children from 6 to 24 months were the most affected by BM. There is a significant difference between cases and controls by age. Children suffering less than six months from BM have twice the risk of death (OR = 2, 14 [1, 13-4, 04]) compared older children. There is a male predominance with a sex ratio of 1.34 of males: females. Sex was not statistically associated with the death of the BM in our series (Table 1).

The number of siblings and overcrowding were strongly associated with BM mortality, with respective odds ratio of 10.18 [3, 18- 32, 61] and 6, 17 [2, 04 to 18, 61]. Almost half of deceased children had more than three siblings (Table 1).

There was no significant difference associated with vaccination. Over 60% of children in our study population were not fully vaccinated. Children hospitalized after five days of illness had a three-fold greater risk of death (OR = 3.74 [1,46- 9,56]) (Table 1).

When clinical presentation was considered, only the existence of impaired consciousness was linked to greater risk of deaths due to the BM in our study (OR = 13 [3.72 to 45.47]). No significant differences were noted on temperature, general condition, the existence of convulsion and meningeal syndrome (Table 2).

The existence of malnutrition was not associated with mortality due to BM in our study.

Results of CSF examinations, revealed the main organism encountered was *Streptococcus pneumoniae* (78.13%), followed by *Neisseria meningitidis* (12.50%). No *Haemophilius* was detected. There was a significant difference between the cases and controls. The meningococcal meningitis was strongly associated with death in our study (OR = 33 [3-361.95] (Table 3).

There were no significant differences based on CSF glucose and CSF protein results from clinical chemistry tests. A low CSF glucose of less than 2.25 mmol / l (20 mg / dl) was encountered in 41% of children in our study population, while 81% had high CSF protein more than 0.4 g / l (Table 3).

### Multiple logistic regression

After adjustment, the factors associated with BM death in our study were the number of siblings over 3 (14,48 [2,53 - 82,95]), crowding (9,31 [1,39 - 62,29]), time before hospitalization more than five days (9,26 [1,36 – 62,92]), altered consciousness (47,74 [6,24 - 364,96]), and when the causative organism is meningococcal meningitis (36,68 [1,90 – 704,97]) (Table 4).

### Survival Analysis

**Figure 1:**
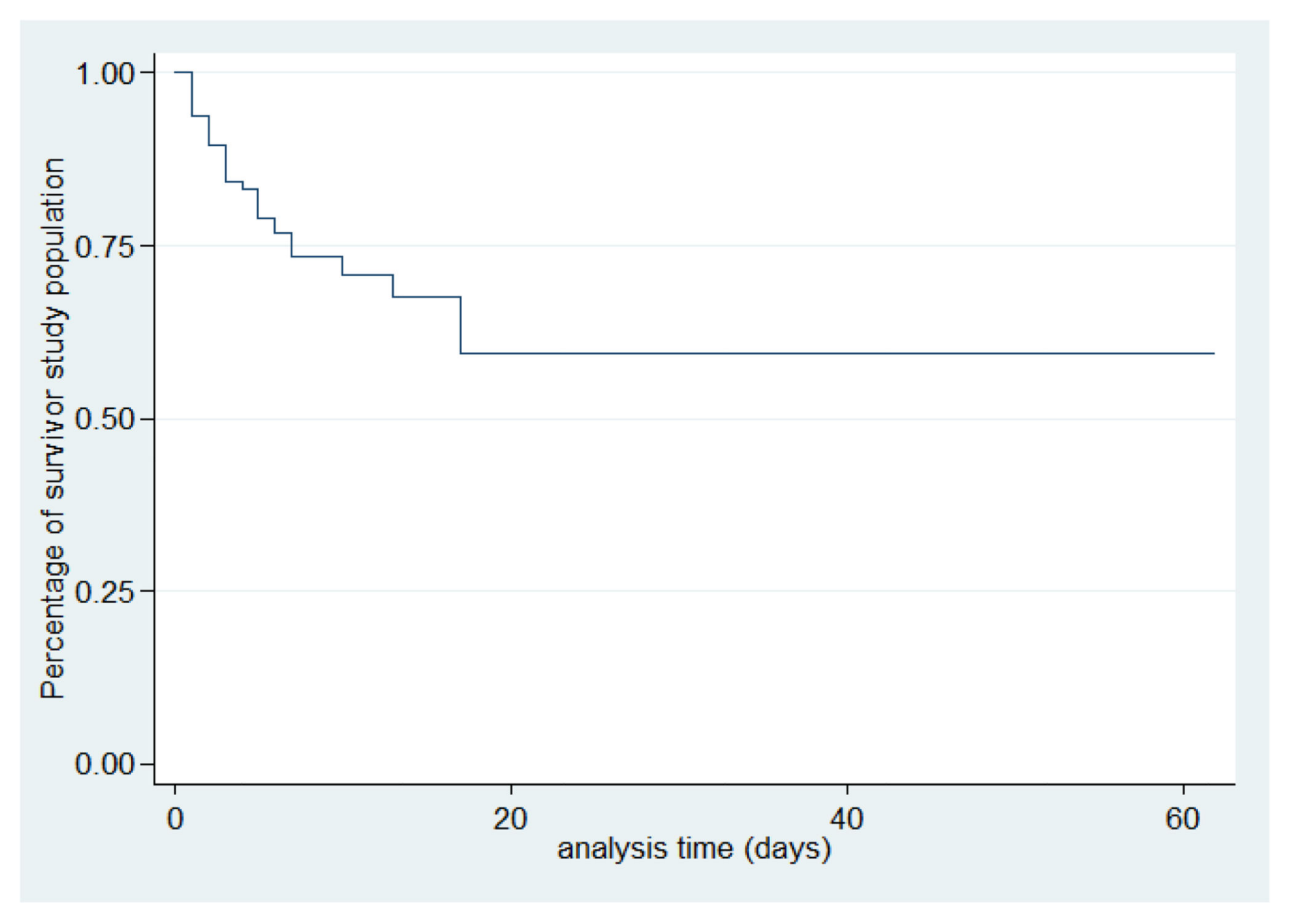
Survival analysis curve of Kaplan-Meier

In our series, the median duration of hospitalization was 11 days with a maximum of 62 days. For the cases, this median duration is 4, 5 days versus 14 days for the controls. There was a gradual decrease in the survival curve; the decline is most important during the first 5 days (Figure 1)

**Figure 2:**
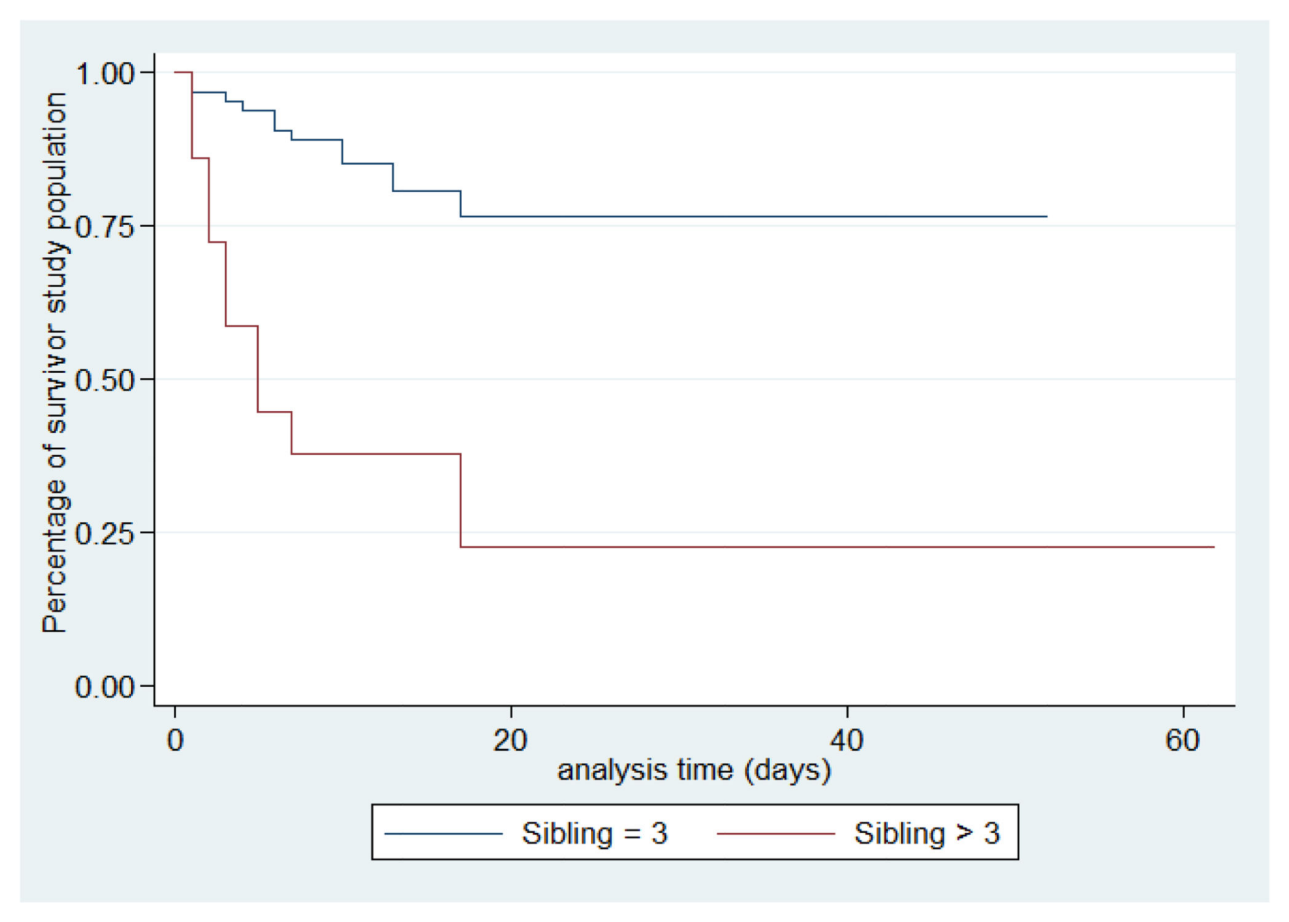
Survival analysis curves according to sibling

**Figure 3:**
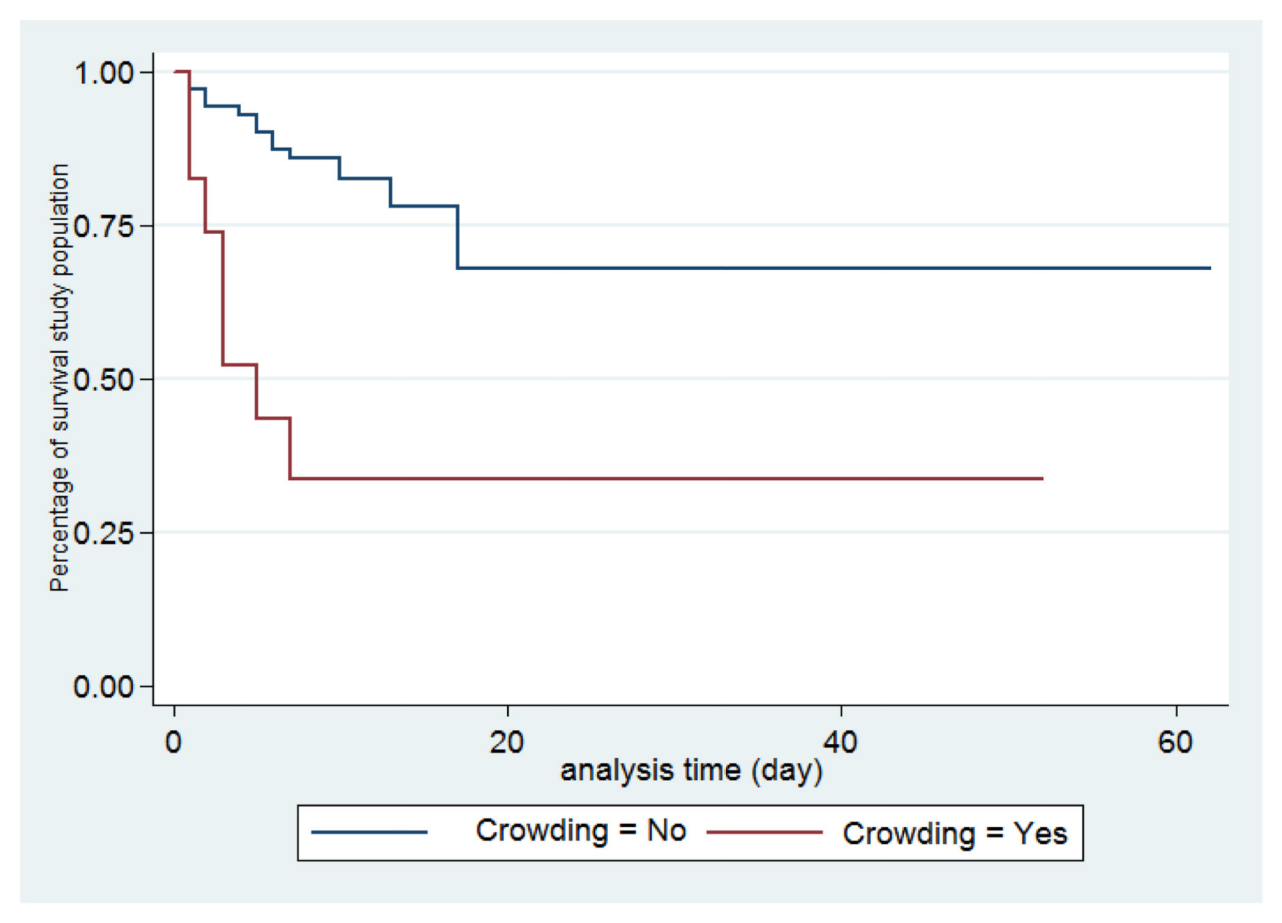
Survival analysis curves according to crowding

**Figure 4:**
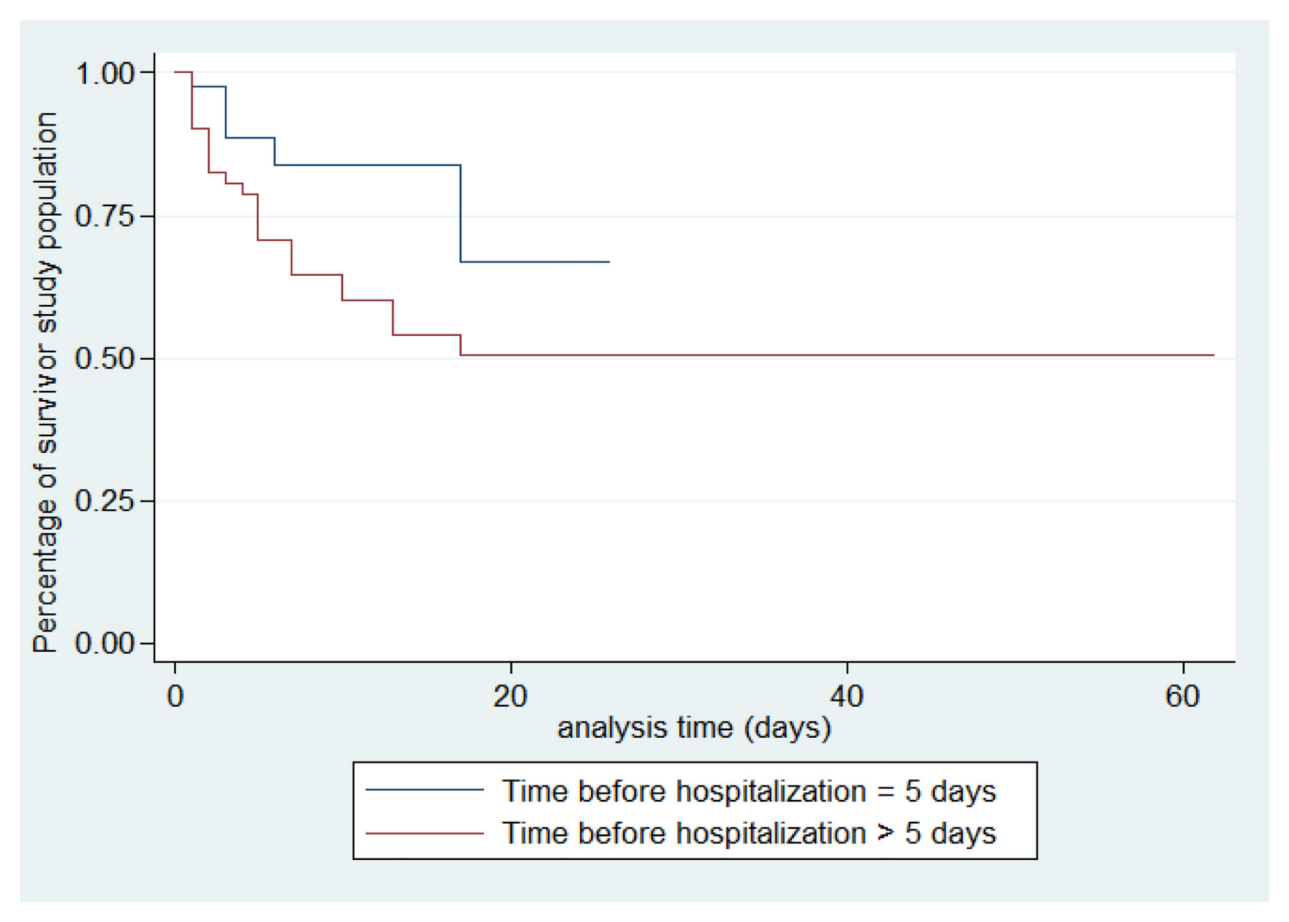
Survival analysis curves according to time before hospitalization

**Figure 5:**
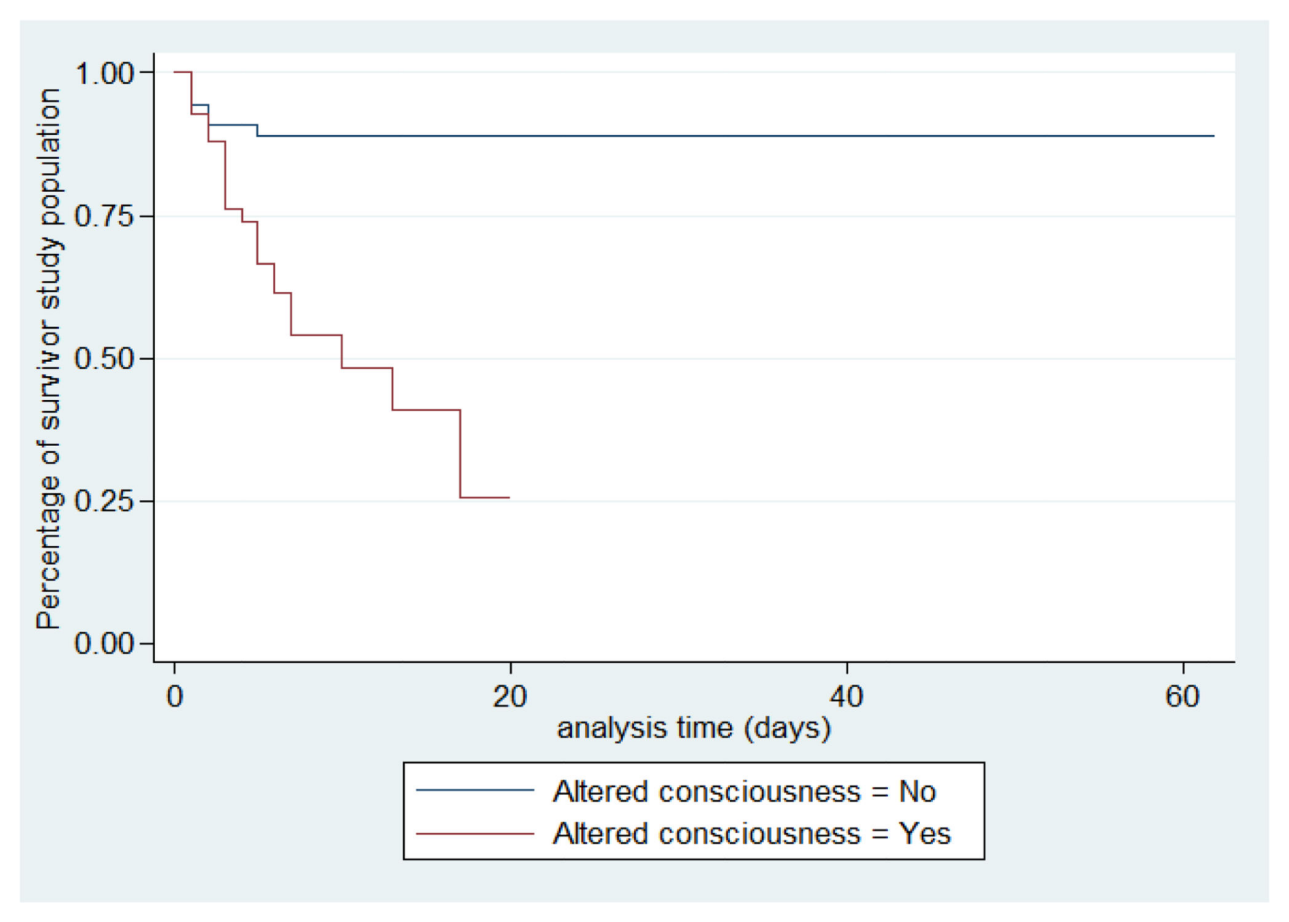
Survival analysis curves according to altered consciousness

**Figure 6:**
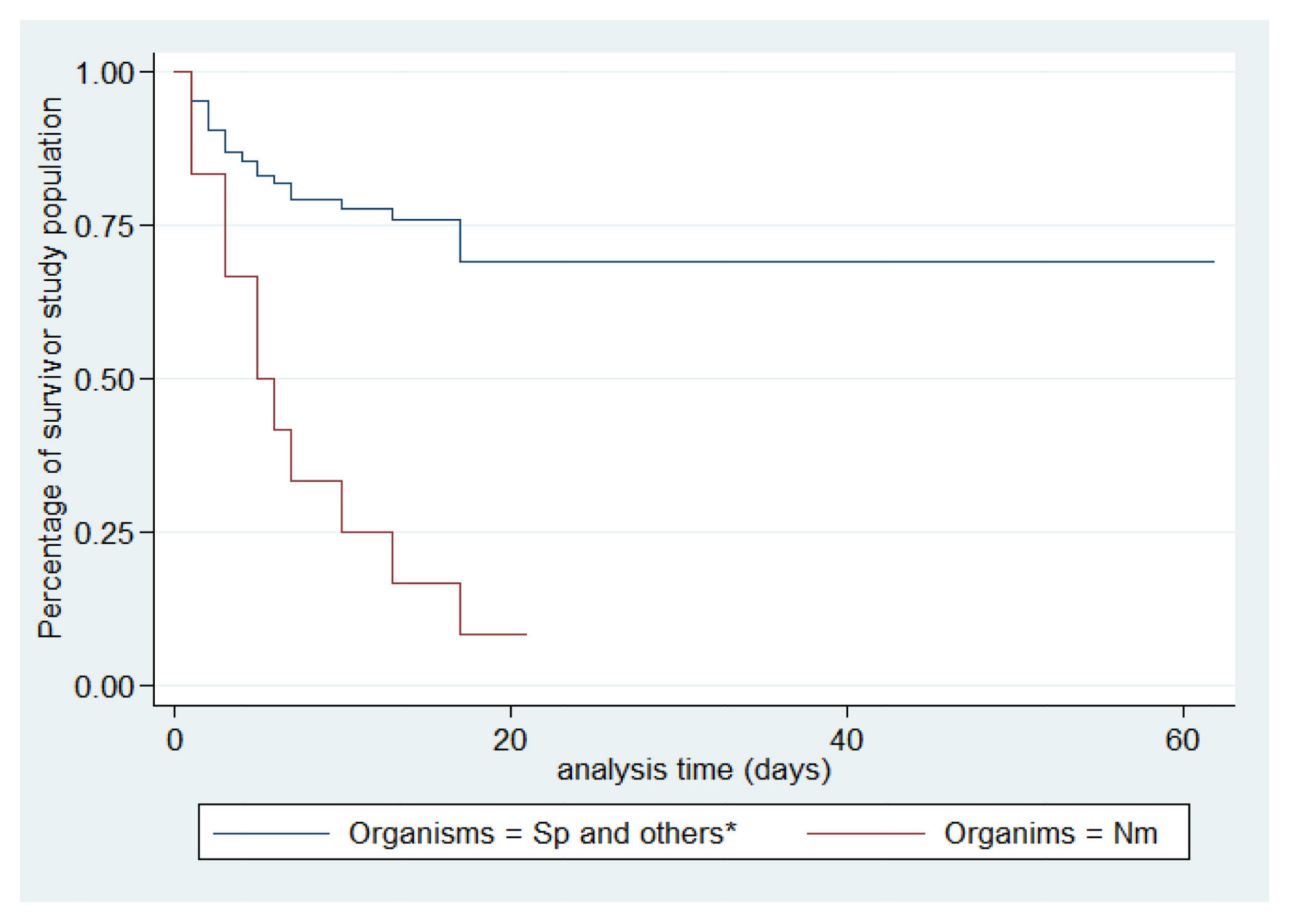
Survival analysis curves according to organisms *Only Streptococcus pneumoniae and Nesseira meningitidis were found in the cases, other germs are Streptococcus B, Staphylococcus aureus and E. coli, no Haemophilius was found.

According to the existence of factors associated with death, survival is shorter if the number of siblings is more than three, with crowding, altered consciousness, and when the causative organism is meningococcal meningitis (Figures 2-6).

## Discussion

The mean childhood BM mortality is around 5% worldwide^1^–^4^ with differences depending on the country and study site. For example it was reported as 28.7% in Malawi, 33% in Angola, and up to 37% in Latin America^7^–^10^. In developed countries, this value is much lower as for example in France with a death rate ranging from 5.9 % to 10.2% over the past decade^11^.

The mortality in our study was 17, 71%, which is lower than for the other African countries, but it could be revised upwards due to the relatively small sample size as a result of exclusion of children with missing data. Also one retrospective study such as ours cannot provide an accurate estimation of the mortality due to BM in Madagascar.

In France, Nigeria, Guatemala and Malaysia, the category mainly affected by BM is children less than 12 months^11^–^14^. Basri in Malaysia shows that children less than 12 months are at increased risk of death due to BM (OR = 3.13 [1.33 to 7.24])^14^. These results are in agreements with our study, children suffering from BM and less than six months of age have twice the risk of death. Young infants are a population at risk for BM death and may require a more tailored support.

The majority of BM impact studies found a male predominance of 50 to 60%, as MacCormick in Malawi^8^, Kuti in Nigeria^12^, and Basri in Malaysia^14^ have reported. The sex ratio depends on the socio-demographic breakdown of each country. However, sex is not a death risk factor related to BM.

Our study found a strong association between the number of siblings and death among children with BM. Our findings thus confirmed what the literature clearly established and reported, an association between the risk of developing BM and the number of siblings^15^, ^16^. However, no study has shown the effect of siblings on the survival of children with BM. In our study, the effect of the number of siblings on the survival of children with BM could be explained by the assumption of difficulty of a hospitalized child from a large family in a developing country. It’s very difficult for parents devoting time because of economic difficulties to take care all their children.

The same applies to overcrowding. Huljer highlights that children staying in a nursery have twice the risk of acquiring BM, but the association with death has not been established. Overcrowding exposes the child to more virulent organisms, and once again, the large family cannot cope with the hospital charge or fee due to demands of the family. This may explain the strong association of deaths and number of of siblings or overcrowding in our study.

In several countries in Latin America, Garcia and his team have demonstrated the protective effect of the haemophilus vaccine on the risk of meningitis, with a relative risk ranging from 0.07 to 0.21^17^. Suarez demonstrates that the efficacy of pneumococcal vaccine on mortality in pneumococcal diseases including meningitis with a decrease of more than 39.4% [14.3 to 57.1] in Lima and 39.6% [19.9 -59.4] in Peru^1^–^8^.

In Madagascar, more advanced studies are needed to demonstrate the relationship between vaccination and BM death. Analyzes should be carried out in the pre- and post-vaccination years.

The delay before admission is strongly associated with BM mortality in our study. According to Olson in Guatemala, children with a delay of more than three days were 3.7 times at greater risk of death (p = 0.003)^13^. This time is proportional to the severity of signs and complications.

Of the clinical presentation signs, only the existence altered consciousness has been identified as a factor associated with the BM death in our study. Several studies emphasize the importance of disorders of consciousness on the survival of children with BM. The association is more or less important depending on the study: 14.4 [9.42 to 22.1] for MacCormick in Malawi; 2.61 [1.44 to 4.72] according to Pelkonen in Angola; 3.31 [1.44 to 7.64] for a Glasgow coma score of less than 8 according to Roine^8^,^9^,^14^. The evaluation of neurological status is primordial during a childhood BM. Any disturbance of consciousness imposes appropriate care in emergency. This is also true for ceberal malaria where an association between coma scores and death from cerebral malaria has also been established (references).

Other neurological signs including seizures are strongly associated with death. Roine, in several Latin American countries shows that children with BM and having convulsions have four times the risk of death (OR = 4.23 [1.75 to 10.2])^10^.

Other vital functions impairment can influence the survival of childhood BM. The presence of respiratory distress is strongly linked to BM death, with an odds ratio of 2.42 [1.17 to 5.03] according to Pelkonen and 6.38 [2.61 to 15.61] according to Roine in Angola^9^,^19^. Similarly, the sign of circulatory collapse is a poor prognostic factor of survival for children with BM, with a risk of 3.08 [1.29 to 6.20] in case of low blood pressure, and 5.11 [1.80 to 14.54] when there is prolonged capillary refill time^12^,^19^. A prospective study with a larger number of participants would be more appropriate to highlight the impact of different clinical parameters on the survival of childhood BM.

Acute malnutrition is a factor associated with death according to Ronie with a risk of 2.55 [1.05 to 6.17] for moderate malnutrition and 5.85 [2.53 to 33.5] for severe malnutrition^8^. MacCormik demonstrates that HIV-positive children with BM were 1.65 [1.20 to 2.21] times higher risk of dying 8. In our study we did not obtain history of maternal HIV positivity. The only workable diagnostic testing in our study was CSF bacteriological and clinical chemistry examination. Streptococcus pneumoniae was the most frequently encountered and it has been shown that meningococcus was linked to BM death.

Although Streptococcus is the most commonly encountered pathogen during BM, the highly offending organisms in BM death vary between studies. It was Salmonella spp for MacCormick in Malawi (OR = 2.11 [1.06 to 4.08]), and Streptococcus according to Roine in Latin America (OR = 5.59 [2.16 to 6.45])^8^,^10^. The variability of the causative agent might be related to the vaccination coverage in each country. The haemophilus vaccine was introduced in Madagascar in 2002, and coverage was estimated to 70% in 2012 and 69% in 2015. Pneumococcal vaccine was introduced in 2010 with an estimated coverage of 76% in 2013 and 69% in 2015. Meningococcal vaccine is not yet part of the Malagasy EPI^20^.

The CSF glucose and protein were not associated with death in our study. A low CSF glucose would be tied to around four times more deaths during a childhood BM according to Roine in Angola (OR = 4.47 [1.72 to 11.01]) and Shinjoh in Japan (OR = 3 65 [2.02 to 6.56])^19^, ^21^. Roine demonstrated in Latin America that children with CSF protein greater than 250 g / dl had a higher risk of death during BM (OR = 2.72 [1.12 to 6.63]).^10^

In our study, it was shown that survival significantly decreases as the presence or absence of factors associated with death and the majority of deaths occur within the first 5 days. According to Roine, the BM median survival time is 18.5 hours, the majority of deaths occur during the first eight hours^19^. This emphasizes the urgency in support of BM. Intensive care should be emphasized especially in the first hours of admission. The present study is limited by a number of factors. Firstly, we did not obtain information on possible concomitant infections of diseases such as severe malaria, influenza or viral diseases, which could have affected the outcome of patients. Presence of a vaccination card does not always mean actual immunization status as is also lack of a card. Despite these limitations and the limited sample size the present study has provided useful results on BM in childhood in Madagascar and possible prognostic markers.

## Conclusion

This study identified the different variables that account for childhood BM deaths at CHUMET, with the aim to improve the care of patients and to reduce the risk of deaths. Factors associated with death from BM identified in this study reinforce those already shown in the literature. These are mainly indicators of low socioeconomic status, severity of clinical signs and particularly virulent organisms. The absolute urgency of addressing this disease has been highlighted by the survival analysis. The detection of patients at risk allows adequate support right from admission. Further studies are necessary, given the limitations of the present study, among other things, the establishment of the childhood BM severity score, and the assessment of care in emergency is particularly essential.

## Figures and Tables

**Table 1:** Univariate analysis of demographic and social settings and medical history for 446 children aged 0 to 59 months old hospitalized with BM at CHUMET, Antananarivo, Madagascar, between 2012 and 2015

Demographic and Social Settings and Medical History	Categories	Status Arm				P	OR	95% CI
		Cases (32)		Controls (64)				
		N	%	n	%			
**Gender**	Male	20	62.5	35	54.69	0.466		
	Female	12	37.5	29	45.31			
**Age**	3-6 months	10	31.25	30	40	0.011*	2.14	1.13-4.04
	7-23 months	14	43.75	32	46		1	
	24-59months	8	25	2	10		0.14	0.03-0.63
**Siblings**	≤3	12	37.5	55	85.94			
	>3	20	62.5	9	14.06	<0.001	10.18	3.18- 32.61
**Crowding**	No	17	53.13	56	87.50			
	Yes	15	46.88	8	12.50	0.000	6.17	2.04-18.61
Vaccine**	complete	10	31.25	20	31.25			
	incomplete	22	68.75	44	68.75	1		
**Time before hospitalisation**	≤5 days	13	40.63	46	71.88			
	>5 days	19	59.38	18	28.13	0.003	3.74	1.46- 9.56

* Fisher exact test

** According to Malagasy EPI(complete = up to date for age)

**Table 2:** Univariate analysis of clinical features and associated pathology and study arm

Clinical features and associated pathology	Categories	Study Arm				P	OR	IC à 95%
		Cases (32)		Controls (64)				
		n	%	n	%			
**General condition**	Good	18	56.25	47	73.44			
	Altered	14	43.75	17	26.56	0.09		
Temperature	Non febrile	2	9.09	2	4.88			
	Febrile	20	90.91	39	95.12	0.606*		
Altered consciousness	No	6	18.75	48	75			
	Yes	26	81.25	16	25	<0.001	13	3.72-45.47
Seizures	No	7	21,88	23	35.94			
	Yes	25	78.13	41	64.06	0.161		
Meningeal syndrome	No	15	48.88	23	56.1			
	Yes	17	53.14	18	43.9	0.434		
Malnutrition	No	24	75	57	89.06			
	Yes	8	25	7	10.94	0.074		

* Fisher exact test

**Table 3:** Univariate analysis of laboratory parameters

CSF exam	Categories	Cases (32)		Controls (64)		P	OR	IC à 95%
		n	%	n	%			
Germs/ Organisms	*N. meningitidis*	11	34.38	1	1.56	<0.001*	33	3.00- 361.95
	S.pneumoniae and others**	21	65.63	63	98.44			
CSF glucose	> 20 mg/dl	16	50	39	60.94	0.307		
	≤ 20 mg/dl	16	50	25	39.06			
CSF protein	≤ 400 mg/l	9	28.13	10	15.63	0.147		
	> 400 mg/l	23	71.88	54	84.38			

* Fisher exact test

** Only *Streptococcus pneumoniae* and *Neisseira meningitidis* were found in the cases, other germs are *Streptococcus B*, *Staphylococcus aureus* and *E. coli, no Haemophilius was found.*

**Table 4:** Adjusted odds ratio of factors associated with BM death

Variables	P	Adjusted OR	95% CI
Age	-		
Sibling	0,003	14,48	2,53- 82,95
Crowding	0,021	9,31	1,39- 62,29
Time before hospitalisation	0,023	9,26	1,36 – 62,92
Altered consciousness	<0,001	47,74	6,24- 364,96
Organism : *Neisseria Meningitidis*	0,017	36,68	1,90 – 704,97
